# Posterior Retroperitoneal Laparoscopic Adrenalectomy: An Anatomical Essay and Surgical Update

**DOI:** 10.3390/cancers16223841

**Published:** 2024-11-15

**Authors:** Bogdan Ovidiu Feciche, Vlad Barbos, Alexandru Big, Daniel Porav-Hodade, Alin Adrian Cumpanas, Silviu Constantin Latcu, Flavia Zara, Alina Cristina Barb, Cristina-Stefania Dumitru, Talida Georgiana Cut, Hossam Ismail, Dorin Novacescu

**Affiliations:** 1 Department of Surgical Disciplines, Discipline of Urology, Faculty of Medicine and Pharmacy, University of Oradea, University Street, No. 1, 410087 Oradea, Romania; feciche.bogdanovidiu@didactic.uoradea.ro; 2 Department of Urology, Emergency County Hospital Oradea, Gheorghe Doja Street, No. 65, 410169 Oradea, Romania; alex_big_jsd@yahoo.ro; 3 Doctoral School, Victor Babes University of Medicine and Pharmacy Timisoara, E. Murgu Square, No. 2, 300041 Timisoara, Romania; silviu.latcu@umft.ro; 4 Department of Urology, George Emil Palade University of Medicine, Pharmacy, Sciences and Technology of Targu-Mures, Gh. Marinescu Street, No. 38, 540142 Targu-Mures, Romania; daniel.porav-hodade@umfst.ro; 5 Department XV, Discipline of Urology, Victor Babes University of Medicine and Pharmacy Timisoara, E. Murgu Square, No. 2, 300041 Timisoara, Romania; cumpanas.alin@umft.ro; 6 Department II of Microscopic Morphology, Victor Babes University of Medicine and Pharmacy Timisoara, E. Murgu Square, No. 2, 300041 Timisoara, Romania; flavia.zara@umft.ro (F.Z.); toma.alina@umft.ro (A.C.B.); cristina-stefania.dumitru@umft.ro (C.-S.D.); novacescu.dorin@umft.ro (D.N.); 7 Department XIII, Discipline of Infectious Diseases, Victor Babes University of Medicine and Pharmacy Timisoara, E. Murgu Square, No. 2, 300041 Timisoara, Romania; talida.cut@umft.ro; 8 Center for Ethics in Human Genetic Identifications, Victor Babes University of Medicine and Pharmacy Timisoara, E. Murgu Square, No. 2, 300041 Timisoara, Romania; 9 Department of Urology, Lausitz Seeland Teaching Hospital, University of Dresden, Maria-Grollmuß-Straße, No. 10, 02977 Hoyerswerda, Germany; drismailhossam@gmail.com

**Keywords:** adrenal cancer, adrenal gland morphology, suprarenal gland histology, retroperitoneum/retroperitoneal topography, abdominal wall anatomy, surgical anatomy, minimally invasive surgery, trocar placement technique, operative protocol

## Abstract

Posterior retroperitoneal laparoscopic adrenalectomy (PRLA) has emerged as a revolutionary, minimally invasive technique for adrenal gland surgery, offering significant advantages over traditional open approaches. This narrative review aims to provide a comprehensive update on PRLA, focusing on its anatomical foundations, surgical technique, and clinical implications. We conducted an extensive review of the current literature and surgical practices to elucidate the key aspects of PRLA. The procedure leverages a unique “backdoor” approach, accessing the adrenal glands through the retroperitoneum, which necessitates a thorough understanding of the posterior abdominal wall and retroperitoneal anatomy. Proper patient selection, meticulous surgical planning, and adherence to key technical principles are paramount for successful outcomes. In this paper, the surgical technique is described step by step, emphasizing critical aspects such as patient positioning, trocar placement, and adrenal dissection. PRLA demonstrates reduced postoperative pain, shorter hospital stays, and faster recovery times compared to open surgery, while maintaining comparable oncological outcomes for appropriately selected cases. However, the technique presents unique challenges, including a confined working space and the need for surgeons to adapt to a posterior anatomical perspective. We conclude that PRLA, in the right clinical setting, offers a safe and effective alternative to traditional adrenalectomy approaches. Future research should focus on expanding indications and refining techniques to further improve patient outcomes.

## Simple Summary

This paper explores a modern surgical technique called posterior retroperitoneal laparoscopic adrenalectomy (PRLA), which is used to remove the adrenal glands. Unlike traditional open surgery, PRLA uses small incisions in the patient’s back, allowing surgeons to access the adrenal glands directly without disturbing other organs in the abdomen. Here, we explain the complex anatomy involved, describe how surgeons perform this procedure step by step, and discuss its benefits and challenges. PRLA can lead to less pain after surgery, shorter hospital stays, and quicker recovery for patients. However, it requires surgeons to have a deep understanding of the body’s structure from a different angle than they are used to. This technique is becoming increasingly popular, but it is not suitable for every patient. Our goal is to help surgeons better understand PRLA, potentially improving outcomes for patients who need adrenal gland surgery.

## 1. Introduction

The adrenal glands, or suprarenal glands, first described in 1552 by Bartholomaeus Eustachius, were not well understood until the 19th century, when Thomas Addison described adrenal insufficiency [1]. The first successful adrenalectomy was performed in the early 1900s [2], marking the beginning of surgical interventions for adrenal diseases. The adrenal glands, located deep within the upper retroperitoneum, historically necessitated large thoraco-abdominal incisions, either anterior or posterior, for access during open procedures. This approach, while effective, was associated with significant morbidity, including substantial postoperative pain, parietal complications, extended hospital stays, and lengthy recovery periods, as well as higher overall complication rates. Almost a century later, the introduction of minimally invasive techniques marked a turning point in adrenal surgery, offering patients the benefits of reduced morbidity and improved outcomes.

Laparoscopic adrenalectomy (LA) has revolutionized the surgical management of adrenal gland disorders, establishing itself as a superior alternative to traditional open surgery. The development of laparoscopic nephrectomy in 1990 paved the way for minimally invasive techniques that revolutionized the field [3]. The first LA was conducted by Go et al. on 17 January 1992, in Japan [4]. The initial publication on this procedure was authored by Higashihara et al. in July 1992 [5]. Subsequently, Gagner et al. reported their first case in November 1992, having performed it in March of the same year [6]. This pioneering procedure quickly gained traction, with several groups publishing case series that emphasized the considerable advantages of this new approach, including shorter postoperative hospital stays, fewer wound complications, reduced pain, and a faster return to normal daily activities [3].

Subsequently, building upon these early groundbreaking surgical developments, numerous studies have further validated the feasibility and efficacy of LA. Although many of these studies were retrospective and involved relatively small patient cohorts, they consistently demonstrated that LA outperformed open surgery in several key areas. Notably, LA was associated with less intraoperative blood loss, shorter hospitalization intervals, and fewer overall complications [7,8]. Initially, the operative times for LA were longer compared to open surgery. However, as surgical teams gained experience with advanced laparoscopic techniques, particularly adrenalectomy, these times became comparable to those of the open approach [9]. Furthermore, LA required fewer postoperative analgesics and facilitated quicker social reintegration, thus reinforcing its current status as the gold standard for adrenal surgery in most patients.

Early on, the anterior transperitoneal LA quickly gained popularity due to its familiar anatomy, ample working space, and versatility in accessing both adrenal glands, with the caveat of requiring patient repositioning [6]. Over the following decades, the transperitoneal approach became the gold standard for the surgical management of most adrenal disorders, including functional adrenal tumors (such as pheochromocytomas, aldosterone- and cortisol-secreting adenomas, etc.), non-functional tumors with risk of malignancy, and isolated adrenal metastases, provided there is no invasion into surrounding tissues [10,11,12].

However, the transperitoneal approach has certain limitations that can impact its suitability for some patients. Entering the deep supero-medial retroperitoneal space anteriorly necessitates the mobilization of adjacent organs, such as the liver, spleen, and pancreas, which can be challenging in the presence of adhesions from prior abdominal surgeries. The intraperitoneal insufflation required for transperitoneal laparoscopy can also cause hemodynamic and respiratory changes that may not be well tolerated by some patients, particularly those with cardiopulmonary comorbidities. Furthermore, bilateral adrenalectomy performed transperitoneally requires repositioning the patient during the procedure, prolonging the operative time and increasing the risk of complications [13].

To address these limitations, the posterior retroperitoneal approach to laparoscopic adrenalectomy (PRLA) was developed in the mid-1990s by Mercan et al. [14] and Walz et al. [15]. By accessing the adrenal glands through the posterior abdominal wall, directly into the retroperitoneal space, PRLA avoids the need to enter the peritoneal cavity or mobilize adjacent organs. This results in several advantages, including shorter operative times, reduced operative complexity, less blood loss and postoperative pain, and faster recovery, compared to the transperitoneal approach [15]. Additionally, PRLA allows for bilateral adrenalectomy without the need for patient repositioning, making it an attractive option for patients with bilateral adrenal disease, such as primary pigmented nodular adrenocortical disease or bilateral pheochromocytomas [16]. Moreover, the posterior retroperitoneal approach, due to its unique patient positioning, even allows for simultaneous bilateral adrenalectomy, with two distinct teams, each operating on a different side, at the same time [17].

Notwithstanding its potential benefits, the adoption of PRLA was initially slow due to several factors. The retroperitoneal anatomy is generally less familiar to many surgeons, and the limited working space can make the procedure technically challenging, especially for those with less experience in minimally invasive adrenal surgery. There were also concerns about the learning curve associated with PRLA and the potential for complications, such as bleeding or injury to adjacent vascular structures.

Over time, however, technical refinements and increasing surgeon experience have helped to overcome these challenges and establish PRLA as a safe and effective alternative to the transperitoneal approach. The most significant contributions to the evolution of PRLA came from Walz et al. in the early 2000s [18], who introduced several modifications to the technique, including the use of higher insufflation pressures (20–25 mmHg) to create a larger working space, as well as a standardized approach to adrenal gland dissection. In their seminal series of 560 PRLAs, Walz et al. demonstrated excellent outcomes, with a low rate of major complications (1.3%), high success rate (98.1%), and applicability to a wide range of adrenal pathologies, including pheochromocytomas, aldosterone-/cortisol-secreting adenomas, and adrenal metastases [18]. Subsequently, larger systematic reviews and meta-analyses [19,20] have corroborated the findings of Walz et al. and provided further evidence supporting the aforementioned operative advantages of PRLA over the transperitoneal approach, with comparable outcomes in terms of complications and success rates [20].

This narrative review aims to provide a comprehensive update on the role of PRLA in modern adrenal surgery, focusing on patient selection, indications, contraindications, and technical considerations, while extensively exploring the intricate surgical anatomy of the adrenal glands and discussing critical retroperitoneal topography and abdominal parietal architecture, emphasizing their practical implications for PRLA. By examining the latest evidence and expert opinions, we seek to guide surgical decision-making and optimize outcomes for patients undergoing adrenalectomy. Through this in-depth exploration of surgical anatomy and operative protocols, we hope to equip surgeons with the knowledge and tools necessary to successfully integrate PRLA into their surgical practice and offer their patients the benefits of this innovative approach to adrenal surgery.

## 2. Adrenalectomy Approach Selection Considerations

As the body of evidence supporting PRLA continues to grow, it has emerged as a valuable option in the armamentarium of minimally invasive adrenal surgery. Its distinct advantages, particularly for patients with bilateral adrenal disease, prior abdominal surgery, or those seeking faster recovery, have solidified its role in the surgical management of adrenal disorders. However, it is crucial for surgeons to have a deep understanding of the indications, contraindications, and technical nuances of PRLA to ensure optimal patient selection and outcomes.

The decision to perform adrenalectomy is based on several factors, primarily categorized into three main indications: First, functional adrenal tumors, regardless of size, generally warrant surgical intervention. These include tumors responsible for primary Cushing’s syndrome (hypersecretion of cortisol) and primary Conn’s syndrome (hypersecretion of aldosterone), as well as pheochromocytomas (catecholamine-producing tumors) [21]. In some cases, bilateral adrenalectomy may be necessary, particularly in certain forms of Cushing’s disease or in multiple endocrine neoplasia (MEN) syndromes [22].

The second category encompasses tumors with suspected or confirmed malignancy. Adrenocortical carcinoma, though rare, presents a significant challenge, with approximately half being non-functional and the remainder secreting various hormones. Malignant pheochromocytomas, accounting for 12–29% of all pheochromocytomas, pose a diagnostic dilemma due to the lack of definitive preoperative imaging or histological criteria for malignancy. Additionally, metastatic tumors in the adrenal glands, commonly in lymphomas or from primary sites such as the lungs, breast, liver, colon, melanomas, and kidneys, may necessitate adrenalectomy [1,22].

The third category involves non-functional tumors with a risk of malignancy, primarily determined by the size of the lesion. A correlation exists between tumor size and malignancy risk, with lesions larger than 6 cm carrying a 25% risk of malignancy [21]. This size-based approach to surgical decision-making has become a crucial factor in the management of incidentally discovered adrenal masses.

Herein, the management of adrenal masses requires a nuanced approach, balancing the risks of malignancy and hormonal dysfunction against the potential morbidity of surgical intervention. While clear guidelines exist for functional tumors and those with high suspicion of malignancy, the management of non-functional incidentalomas remains a subject of ongoing research and debate in the endocrine surgery community [1].

In contemporary practice, the choice between PRLA and other adrenalectomy approaches depends on a careful consideration of patient factors, tumor characteristics, and surgeon experience. A thorough preoperative evaluation, including hormonal testing, high-resolution imaging, and assessment of comorbidities, is essential to guide surgical decision-making [23]. Ultimately, the goal is to select the approach that offers the best balance of safety, efficacy, and patient outcomes.

Advances in radiological imaging have increased the detection of adrenal incidentalomas—adrenal masses ≥ 1 cm in diameter discovered incidentally during imaging for other conditions. The prevalence of adrenal incidentalomas is approximately 2%, increasing with age to about 4% in middle-aged individuals and 10% in the elderly [24]. Importantly, ~90% of adrenocortical tumors are still >4 cm at diagnosis [24]. Thus, tumors larger than 4 cm or those that enlarge by 1 cm during the observation period typically warrant surgical removal following thorough endocrine evaluation [24].

Minimally invasive surgery, particularly PRLA, has significantly impacted the management of these tumors, improving patient outcomes without altering the fundamental indications and goals of treatment. However, it is important to recognize that PRLA may not be suitable for all patients or all adrenal pathologies [25]. Large tumors (>6–8 cm) or those with suspected malignancy may be better served by an open or transperitoneal approach, which allows for wider resection margins and minimizes the risk of tumor spillage [26]. Conversely, patients with extensive prior abdominal surgery or severe cardiopulmonary comorbidities may not be ideal candidates for anterior transperitoneal LA, due to intraperitoneal adhesions, which make it harder to tolerate transperitoneal insufflation pressure, as opposed to retroperitoneal [27].

The choice between laparoscopic and open adrenalectomy should be guided by a thorough evaluation of each case, considering factors such as tumor size, location, and the patient’s overall health. A multidisciplinary approach, involving surgery, radiology, anesthesiology, and endocrinology, is essential to establish the best surgical strategy and ensure the best possible outcomes for patients. While LA has become the gold standard for most adrenal surgeries, open surgery remains a vital option for complex cases where minimally invasive techniques may not be feasible.

Despite the solid advantages of LA, there remain specific indications for open adrenalectomy. Open surgery is often preferred for malignant adrenal tumors, especially those >12 cm or involving neighboring structures, due to the fragility of such tumors and the risk of iatrogenic disease dissemination. Additionally, patients with significant cardiopulmonary issues, coagulopathy, or extensive previous abdominal or retroperitoneal surgeries may be better suited for open surgery. Conversion to open surgery during PRLA is a rare but necessary event, typically occurring in cases of clinically unforeseen intraoperative findings, such as the detection of more extensive local invasion or regional lymph node metastasis, or due to the inability to control intraoperative complications laparoscopically, i.e., aggressive bleeding due to surgical injuries to major vessels and/or inadvertent injury of surrounding organs [8]. The surgical team’s experience, hospital volume, and a multidisciplinary approach to patient management are critical factors in minimizing the need for conversion and ensuring optimal outcomes.

Ultimately, the indications for PRLA, like other forms of LA, vary depending on the surgeon’s expertise and the specific characteristics of the tumor. While larger tumors, malignant tumors, and pheochromocytomas present greater challenges, they are not absolute contraindications for PRLA. According to the Society of American Gastrointestinal and Endoscopic Surgeons (SAGES) guidelines, tumors ≥ 7.5 cm pose increased risks, including longer surgeries, more blood loss, extended hospital stays, and higher conversion rates to open surgery [10]. However, for small adrenocortical cancers and non-invasive metastatic adrenal tumors, the European Society for Medical Oncology (ESMO) guidelines recommend LA [28]. Studies have shown that outcomes for LA and open surgery in these cases are similar [28]. However, it is worth highlighting the importance of adhering to oncological safety surgical principles and converting to open surgery if necessary.

Contraindications to PRLA can be categorized into relative and absolute contraindications, centered around both patient factors and tumor characteristics. Patient body habitus plays a crucial role, with a body mass index (BMI) > 40 being generally considered a relative contraindication due to the limited working space in the retroperitoneum. Similarly, inadequate distance between the 12th rib and the posterior superior iliac crest can impede proper access and instrument maneuverability, potentially precluding the use of this approach. Tumor size and nature also factor into the decision-making process. Lesions larger than 7 cm pose challenges within the confined retroperitoneal space, while any suspicion of malignancy with potential invasion into surrounding structures may necessitate a more extensive surgical approach [25,29,30].

Absolute contraindications include the patient’s inability to tolerate general anesthesia or maintain a prone position during the procedure, both of which are fundamental requirements for PRLA [1,31]. Confirmed invasion of the tumor into adjacent structures or extremely large tumors that cannot be safely manipulated within the retroperitoneal space also absolutely contraindicate this approach. It is important to note that these contraindications can sometimes be relative, depending on the surgeon’s experience and specific patient circumstances. Some highly experienced surgeons might consider attempting PRLA in borderline cases, such as patients with a BMI slightly >40 or tumors marginally >7 cm, if other factors are favorable [18]. Additionally, while not an absolute contraindication, a history of extensive abdominal surgery may complicate the procedure due to potential adhesions and altered anatomy.

These contraindications underscore the importance of meticulous patient selection for PRLA to ensure optimal surgical outcomes and patient safety. As with any surgical technique, the decision to proceed with PRLA must be made on a case-by-case basis, carefully weighing the potential benefits against the risks and considering alternative approaches when necessary.

## 3. Comparative Morphology of the Adrenal Glands

The adrenal glands, also known as suprarenal glands—the targets of this surgical approach—are crucial endocrine organs with complex functions and an intricate, asymmetrical anatomy. Thus, these glands play a crucial role in secreting multiple distinct hormones, both cholesterol-/lipid- and protein-derived, essential for maintaining bodily homeostasis.

### 3.1. General Histological Structure

**Figure 1:**
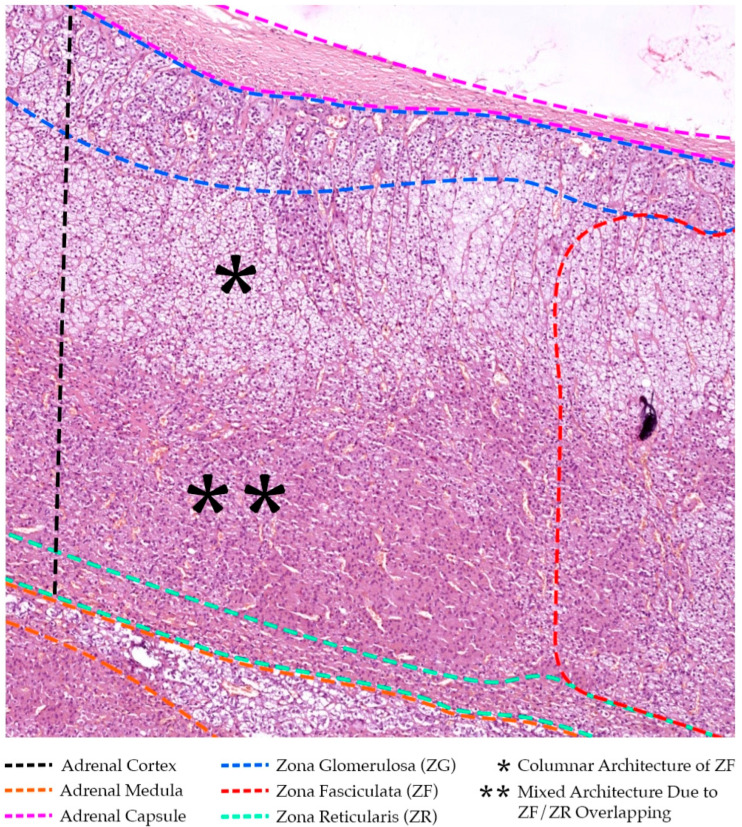
Histological architecture of the adrenal glands (hematoxylin–eosin, 100×).

Herein, the adrenals consist of two histologically and functionally distinct tissue layers: the outer cortex (i.e., the superficial layer), and the central medulla (i.e., the inner deep layer), each wityh different embryological origins and producing different specialized hormones (see Figure 1).

The adrenal cortex is derived from mesenchymal cells attached to the coelomic cavity, adjacent to the urogenital ridge, proximal to the developing kidney, starting around the fourth week of intrauterine life. It secretes steroid hormones from three distinct zones of selectively specialized cortical tissue (see Figure 1), organized from external to internal: mineralocorticoids (e.g., aldosterone) in the zona glomerulosa (15–20% of the whole adrenal cortex), glucocorticoids (e.g., cortisol/cortisone) in the zona fasciculata (60–70%), and androgens (e.g., dehydroepiandrosterone—DHEA) in the zona reticularis (10–15%) [32].

These hormones regulate essential metabolic processes, such as the following: (1) aldosterone—hydroelectrolytic balance and blood pressure regulation; (2) cortisol—glucose metabolism homeostasis, through gluconeogenesis following proteic and lipidic lysis, and immune response modulation, through inflammation suppression; (3) DHEA—precursor androgen for the synthesis of other steroids (estrogen, progesterone, testosterone, and cortisol), which will be converted to fully functional sex hormones in the gonads or other target organs, playing a role in the development of secondary sexual traits and, thus, mitigating sexual function [32,33].

Conversely, the adrenal medulla, originating from ectodermal neural crest cells, represents an integral part of the sympathetic nervous system (i.e., considered to be a specialized sympathetic ganglion, yet lacking distinct synapses and releasing its secretion directly into the bloodstream). These ectodermal neural crest cells migrate to the cortex in the seventh week of gestation and gradually invade it, differentiating into chromaffin cells in response to cortisol, and ultimately forming the medulla centrally, supplied by plexus arteries and capillaries penetrating the cortex and constituting the cortico-medullary portal system. Thus, although lacking a clear histological border with the cortex, the adrenal medulla accounts for ~10% of the total adrenal gland volume (see Figure 1) [33].

Further migration of these adreno-medullary cells—called chromaffin cells due to their brown-staining cytoplasmic granules, seen with chromium salts—as (nor)epinephrine oxidizes to melanin, explains the existence of ectopic adrenal tissues, often located near the aorta and vertebral column, i.e., paraganglia—chromaffin cell clusters distributed on both sides of the aorta. The organ of Zuckerkandl, positioned near the inferior mesenteric artery, represents the largest accumulation of chromaffin cells outside the adrenal medulla. This organ serves as the primary source of catecholamines during the first year of life [34].

Preganglionic sympathetic neurons, which regulate the adrenal medulla, receive synaptic inputs from neurons in the pons, medulla, and hypothalamus. This neuronal connection allows the brain to control sympathetic activity. The medulla responds to sympathetic preganglionic neuron stimulation (i.e., thoracic preganglionic fibers originating at the level of T5-T11) by secreting tyrosine-derived catecholamines (e.g., epinephrine and norepinephrine, dopamine), which are neurotransmitters that are critical for rapid stress responses (i.e., the body’s fight-or-flight response) [33].

### 3.2. Bilateral Gross Anatomy

Macroscopically, these suprarenal glands have a golden yellow–grayish appearance, due to the high contents of intracellular lipids within the cortex, and a firm consistency. Encased posterolaterally by the 10th–12th ribs on the left, and by the 11th–12th ribs on the right, the surprarenals typically weigh 4–8 g, measuring 4–5 cm in length and 2–4 cm in width, in adults. In contrast, they are proportionally larger in newborns, constituting approximately 20–25% of the total body weight [32].

Anatomically, these small (yet vital), triangular-shaped, paired endocrine glands are located deep in the supero-posterior abdomen (projected anteriorly at the level of the epigastrium), nestled within the uppermost part of the retroperitoneum, immediately above the kidneys. Their name, derived from the Latin “ad renalis”, meaning “near the kidney”, aptly describes their location, namely, superiorly and antero-medially along the upper poles of the kidneys, within the paravertebral gutters, flanking the vertebral column at the level of T12 (T11-L1) bilaterally [34]. However, the precise shape and location of each adrenal gland differ slightly from one side to the other. This asymmetry in location and shape is an important consideration for surgeons as they plan and execute their approach.

Thus, as does the right kidney compared to the left, the right adrenal lies lower and more medial to the spine than the left adrenal. Moreover, the right adrenal gland is also thicker and taller, with a more pyramidal shape, being positioned posterior to the inferior vena cava (IVC), within the angle between the right lobe of the liver and the right crus of the diaphragm, with its anterior surface abutting the posterolateral surface of the retro-hepatic IVC, separated by only a thin layer of fascial connective tissue [1,32]. Thus, the IVC isolates the anterior surface of the right adrenal from the more anterior Winslow foramen, the second duodenal segment, and the cephalic pancreas. Lying anterior to the diaphragm and lateral to the right diaphragmatic crus, it is usually covered antero-superiorly and supero-laterally by the “bare area” of the liver or the right hepatic lobe, respectively [35], whereas infero-laterally, the anterior surface may at times be covered by the peritoneum, the liver, and hepatic flexure of the colon [36]. The anterior aspect of the right adrenal may also, albeit rarely, come into contact with the first segment of the duodenum inferiorly. The posterior surface of the right adrenal is divided by a ridge, nestled against the diaphragm superiorly, and against the supero-medial upper pole of the ipsilateral kidney inferiorly. Due to its high suprarenal position, the right adrenal characteristically does not reach the ipsilateral renal hilum, lacking proximity to the right renal vessels [1].

Comparatively, the left adrenal is larger, with a more flattened, semilunar/crescent shape, and lies more antero-medial than superior in relation to the ipsilateral kidney, as compared to the right side [32]. Contrastingly, as it lies partially in front of the left kidney, the left adrenal plunges anteriorly towards the renal hilum, being in close proximity to the left renal vessels with its inferior aspect. Furthermore, the left adrenal is situated posterolateral to the abdominal aorta (~7 mm away), anterior to the diaphragm, with its posterior surface in contact with the left diaphragmatic crus, yet posterior to the stomach, spleen/splenic vessels, and pancreas, and superior—albeit antero-medially—to the upper pole of the left kidney [37]. Superiorly, its anterior surface is closely related to the posterior peritoneal wall of the omental bursa (lesser sac), which, in turn, separates the gland from both the spleen and the cardia of the stomach [32,37]. The inferior portion of the anterior surface, however, lacks peritoneal coverage. Instead, it is in direct contact with the body and tail of the pancreas, as well as the splenic blood vessels [37].

Perirenal fatty tissue surrounds the adrenals bilaterally, both being enclosed within the ipsilateral perirenal fascia, with the exception of the connective tissue segment separating them from the kidneys [32]. This shared fascial envelope is a key anatomical feature that surgeons exploit during PRLA, as the perirenal compartment helps define the surgical plane of dissection. Positioned immediately beneath the diaphragm, the adrenal glands are anchored to the diaphragmatic crura via the renal fascia. Fibrous tissue bands attach the adrenals to the abdominal wall/diaphragm [32]. Thus, it is worth noting that the aforementioned connective tissue segment that separates the adrenal gland from the kidney also becomes important during the dissection phase of the surgery.

These complex anatomical relationships underscore the importance of a thorough understanding of regional anatomy when performing PRLA. The surgeon must navigate carefully around these structures to safely access and remove the adrenal gland.

### 3.3. Vascularization, Lymphatics, and Innervation

**Figure 2:**
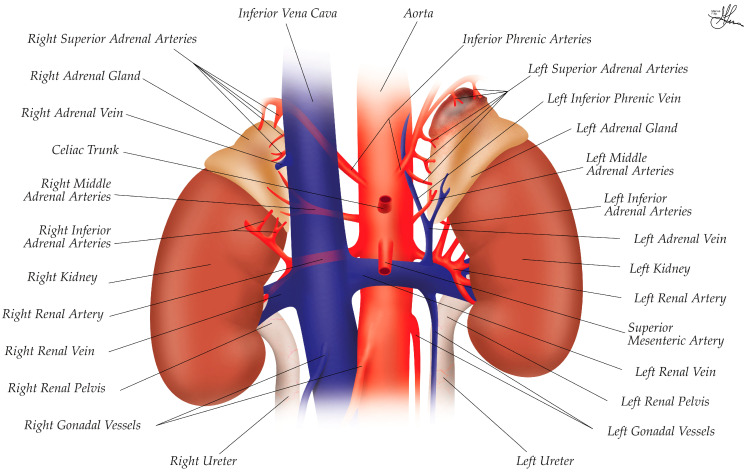
Illustration of bilateral adrenal gland vascularization: anterior view, coronal section.

The vascular supply of the adrenal glands is as complex and asymmetrical as their location. Among the most highly perfused organs, the adrenal glands receive 2000 mL/kg/min of the circulating blood volume, ranking third in perfusion after the thyroid and kidneys [38]. Thus, the adrenal glands receive this rich blood supply from three main homologous arteries on each side, forming a dense arterial subcapsular plexus around the gland. As shown in Figure 2, these are as follows:

1. The superior adrenal arteries: Usually one to three, but up to six to eight per side, the superior adrenal arterial branches arise from the inferior phrenic arteries, before they distribute to the diaphragm, and supply the upper part of the glands.
2. The middle adrenal arteries: Though inconsistent—i.e., single, multiple, or absent—they supply the perirenal fat only, emerging directly from the abdominal aorta, just proximal to the origin of the renal artery. Reaching the inner side of the adrenal, the middle adrenal artery gives branches on both glandular surfaces. On the right side, these branches cross the IVC in a retro-caval manner.
3. The inferior adrenal arteries: One, or at times multiple, these inferior braches arise from the main renal artery, or an accessory or superior polar renal artery, with small collaterals even originating in the superior ureteric artery, and enter the gland through its inferior surface [32,33,34].

In addition to these main sources, the adrenal glands may also receive blood supply from small arteries originating from the subcostal and gonadal vessels. Herein, the main branches divide before entering the glandular tissue and ramify [32]. There may be up to 50 arterioles creating an intricate network under the adrenal capsule, i.e., the subcapsular arterial plexus [1]. Thereafter, sinusoids draw their origin and descend inward, around clusters of glomerulosa cells, in between the zona fasciculata columns, to form a deep capillary plexus in the innermost zona reticularis of the adrenal cortex (see Figure 1). This plexus will provide drainage to the medullary veins [1]. This abundant vascularity is a double-edged sword for surgeons—while it ensures the vitality of the gland, it also increases the risk of troublesome bleeding during surgery if vessels are inadvertently injured.

Venous drainage of the adrenal glands is also asymmetrical and is not correspondent to the arterial system. At the level of the gland’s hilum, a singular consolidated adrenal vein emerges, draining the entire gland. Herein, as the left adrenal vein emerges from the hilum and descends along the anterior surface of the left adrenal gland, it is joined by the left inferior phrenic vein, before ultimately emptying into the left renal vein (see Figure 2). This arrangement creates a longer path for venous drainage on the left side. In contrast, the right adrenal vein follows a more direct route. After emerging from the hilum, it courses obliquely and drains directly into the posterior aspect of the IVC (see Figure 2). This shorter, more direct path can present unique challenges during surgical procedures [32,33,34,39].

In summary, the right suprarenal vein is typically short (~5 mm in length) and drains directly into the IVC on its posterolateral aspect, whereas the left suprarenal vein is longer (~3 cm) and drains into the left renal vein or occasionally into the inferior phrenic vein (see Figure 2) [24]. Adding to this complexity, variations in venous anatomy—particularly on the right side, where the short adrenal vein can be challenging to dissect and control—can affect the surgical approach and technique.

In fact, anatomical variations in adrenal venous drainage are not uncommon (in ~12.8% of patients), especially on the right side. The right adrenal vein may drain into a posterior hepatic vein (1.6% of cases), into the IVC just below a hepatic vein (6.3% of cases), or there may be two adrenal veins draining into the IVC (3.1% of cases). When an adrenal shows two distinct venous trunks at the level of the hilum, they are usually disposed as follows: one main trunk, following the conventional aforementioned course, and on accessory trunks, which drain into the inferior phrenic vein. Occasionally, the right adrenal vein may drain into the IVC immediately above the renal vein [39]. These variations underscore the need for careful preoperative imaging and intraoperative vigilance.

The lymphatic drainage of the adrenal glands is also worth noting. Two lymphatic plexuses exist within each gland—one beneath the capsule and another inside the medulla. These plexuses channel lymph into several node groups: renal hilum nodes, para-aortic nodes (specifically those proximal to the diaphragmatic crux/renal artery), and paracaval nodes. Some lymphatic vessels even traverse the diaphragm via the minute orifices housing splanchnic nerves, thus draining towards the thoracic duct, as well as the prevertebral/dorsal mediastinal lymph nodes [40]. This complex lymphatic drainage system explains the pattern of distant dissemination sometimes seen in adrenal cancer [32].

Relative to their size, the adrenal glands have the largest autonomic supply out of all human organs. Their innervation is derived from multiple sources: the celiac and renal plexuses, and the thoracic splanchnic nerves. These nerve fibers form the suprarenal plexus, which can be further divided into three secondary plexuses: the adreno-celiac, adreno-renal, and adreno-diaphragmatic plexuses. This rich innervation disposes itself between the inner aspect of each gland and the medial celiac/aorto-renal ganglia. It consists mainly of preganglionic sympathetic fibers, which permeate deep within the gland, to finally synapse with the medullary chromaffin cells [40]. This conformation is essential for the rapid secretion of catecholamines in response to stress. Albeit in smaller proportion, post-ganglionic sympathetic fibers also exist, to innervate the cortical blood vessels [32,33,34].

### 3.4. Surgical Implications for PRLA

Vascular control is a critical step in PRLA. Early ligation of the adrenal vein is important to prevent the release of catecholamines. From a posterior perspective, the right adrenal gland presents unique anatomical considerations. The right adrenal vein, a critical structure for ligation during adrenalectomy, is situated posterior to the gland, between it and the IVC. This positioning requires meticulous dissection/mobilization of the right suprarenal gland to ensure proper identification/ligation of the vein. Conversely, the left suprarenal gland offers a somewhat more straightforward approach. The left adrenal vein is typically identified at the infero-medial border of the gland, generally allowing for easier dissection and ligation compared to its right-sided counterpart. However, the left adrenal’s position relative to the kidney introduces its own complexities [41].

Exposure of the adrenal gland requires the mobilization of perirenal fat, identification of the upper pole of the kidney as an initial landmark, and medial/caudal retraction of the kidney to expose the adrenal gland. The left adrenal gland falls in front of the upper pole antero-medially, descending towards the renal hilum, and thus necessitates more extensive mobilization of the left kidney as compared to the right. This anatomical arrangement has significant implications for surgical technique and approach [32,41].

Complete mobilization of the adrenal gland requires attention to its anatomical attachments. Medially, it must be dissected from the diaphragmatic crus. Laterally, it is separated from the kidney. Superiorly, attachments to the diaphragm must be divided. Anteriorly, careful separation from surrounding structures such as the liver or pancreas is necessary. The plane between the adrenal gland and the kidney is typically avascular, allowing for preservation of the renal vasculature [31].

Several anatomical factors can present challenges during PRLA. Obesity can obscure landmarks and limit the working space due to excess retroperitoneal fat. Previous retroperitoneal surgery may result in altered anatomy and scarring. Large tumors can distort the normal anatomy and further restrict the already limited working space. Right-sided tumors present a particular challenge due to their proximity to the IVC, increasing the risk of vascular injury. Pheochromocytomas, with their increased vascularity and friable tissue, require extra caution [1,3].

Common anatomical pitfalls to avoid during PRLA include injury to the diaphragm or pleura during superior dissection, inadvertent entry into the peritoneal cavity, injury to renal vessels during adrenal dissection, avulsion of the short right adrenal vein, and injury to surrounding organs such as the liver, pancreas, or spleen [3].

Understanding the detailed anatomy of the adrenal glands and their surrounding structures is essential for effective and safe surgical interventions, particularly in procedures like PRLA. Mastery of these anatomical nuances enhances surgical precision and patient outcomes. The adrenal glands are vital endocrine organs that play a significant role in the body’s hormonal regulation. Their intricate vascular, lymphatic, and neural networks, coupled with their distinct anatomical positions and relationships with adjacent structures, require thorough knowledge and precision in surgical practices. The transition to a posterior anatomical view in PRLA represents a significant shift from traditional approaches, emphasizing the need for a comprehensive understanding of the adrenal glands’ anatomy to ensure successful outcomes in adrenal surgery.

## 4. Relevant Surgical Topography and Anatomical Landmarks for PRLA

PRLA constitutes a unique approach to adrenal gland removal that challenges traditional surgical perspectives. At its core, PRLA represents a so-called “backdoor” approach [18], which quite literally rotates the conventional adrenal surgery overview 180 degrees. Unlike the more familiar transperitoneal laparoscopic technique, the fundamental premise of PRLA involves positioning the patient prone, with trocar access incisions being made posteriorly, bypassing the peritoneal cavity entirely and facilitating direct access to the adrenals. However, this seemingly simple change in orientation brings with it a plethora of anatomical considerations that surgeons must grapple with. Thus, while the adrenal anatomy remains consistent regardless of approach, surgeons must adapt to a more confined working space and shift their mental model to a posterior anatomical view—a perspective that diverges significantly from traditional surgical training. Despite its advantages, the unfamiliarity of surgeons with the posterior anatomical perspective has thus far been the main limiting factor regarding the widespread adoption of PRLA [15].

Even so, PRLA offers significant benefits over traditional approaches, provided the surgeon has a thorough understanding of the complex anatomy of the adrenal glands and retroperitoneal space. The reduced operative time, less postoperative pain, and minimized risk of intra-abdominal complications make PRLA an attractive option. However, the limited working space and the need for anatomical orientation pose challenges. Surgeons must adapt to the posterior perspective, which can be challenging without sufficient anatomical familiarity. This section discusses critical retroperitoneal topography and abdominal parietal architecture, emphasizing their practical implications for PRLA.

### 4.1. Parietal Anatomy for Trocar Placement

The anatomical positioning of the adrenal gland determines the placement of trocars in the “backdoor” approach. Thus, the efficacy of PRLA is contingent upon precise trocar placement, which necessitates a comprehensive understanding of the abdominal wall’s layered anatomy. The abdominal cavity is bordered by three anatomically distinct walls: superior (the diaphragm), posterior, and antero-lateral.

**Figure 3:**
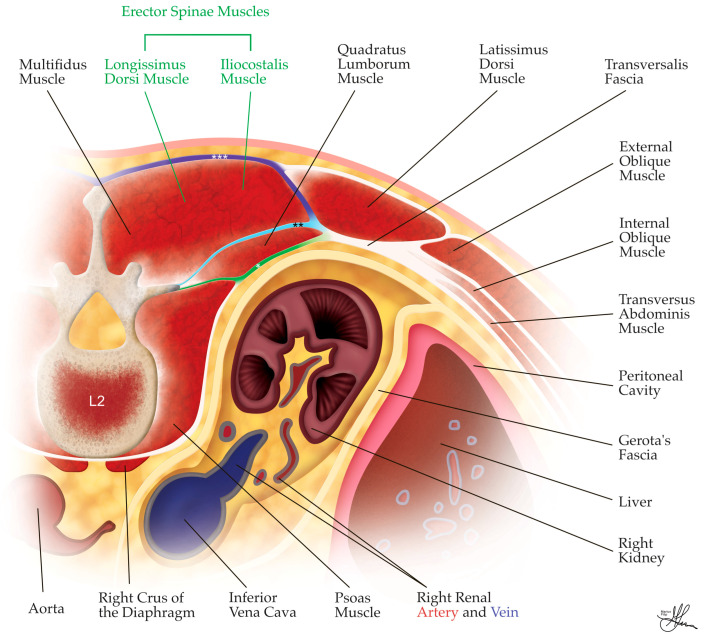
Abdominal parietal anatomy illustration, focusing on the right posterior and antero-lateral walls, in an axial L2 section of a prone patient, superior view. *—Anterior layer of thoracolumbar fascia (marked in green); **—middle layer of thoracolumbar fascia (marked in light blue); ***—posterior layer of thoracolumbar fascia (marked in deep blue).

The antero-lateral abdominal wall comprises the rectus abdominis, the pyramidalis, the transversus abdominis (TA), the internal oblique (IO), and the external oblique (EO) muscles. During PRLA, surgical access only involves the posterior half of the antero-lateral abdominal wall. As depicted in Figure 3, this posterior half area includes three large muscles (from superficial to deep): the EO, IO, and TA muscles, separated individually by thin layers of connective tissue. The transversalis fascia nearly completely covers the deep surface of the TA muscle. Anteriorly, these three muscles terminate through a common aponeurosis, which distributes itself to encase the rectus muscle and form the linea alba anteriorly, at the midline [42].

**Figure 4:**
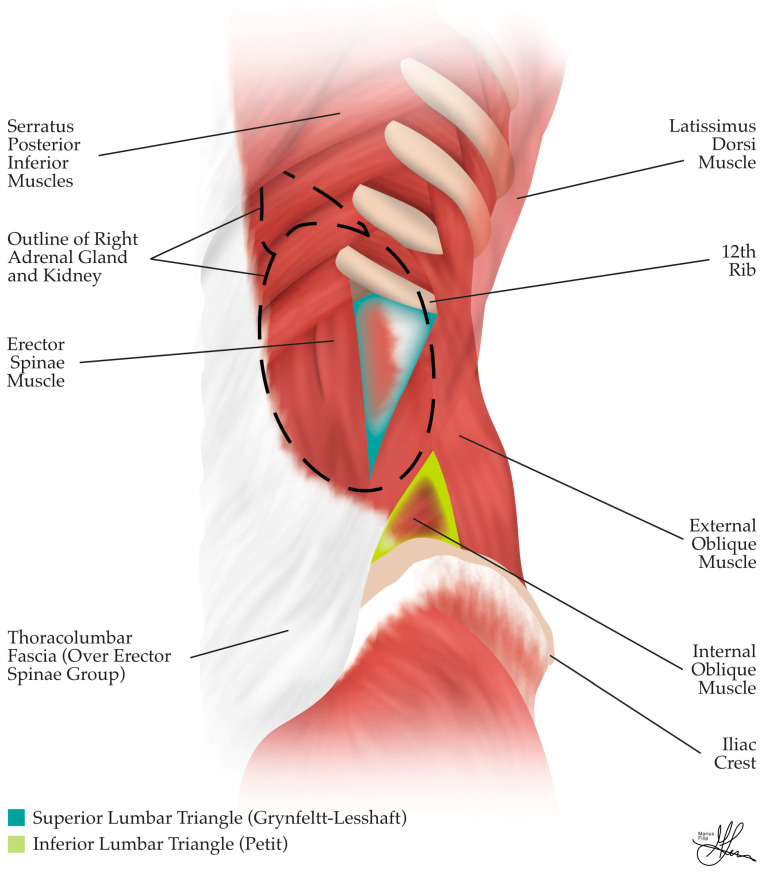
Posterior abdominal wall anatomy illustration: right side, posterior view, coronal section.

The anatomy of the posterior abdominal wall is another crucial consideration in PRLA. Herein, this posterior wall includes the spine and two lumbo-iliac regions, symmetric bilaterally, bordered cranially by the 12th rib, laterally by the lateral border of the quadratus lumborum, caudally by the iliac crest, and medially by the spine (see Figure 4). This area contains three groups of muscles and serves as the medial landmark for performing PRLA. These muscle groups are described, from dorsal to ventral, as follows:

1. The dorsal group comprises the latissimus dorsi muscle and its aponeurosis (see Figure 3), the serratus posterior inferior muscle (see Figure 4), and the erector spinae (ES) muscle group, i.e., comprising the longissimus dorsi and iliocostalis muscles in the lumbar region (see Figure 3).
2. The middle group consists of the dorsal insertion of the TA muscle aponeurosis and the intertransverse process muscles (i.e., intertransversarii, transversospinales/multifidus muscle—see Figure 3).
3. The ventral group, located ventral to the TA aponeurosis, includes the quadratus lumborum and the psoas muscles (see Figure 3) [32,42].

During PRLA, trocar placement occurs lateral to the lumbo-iliac region, with the lateral border of the ES muscle group, in particular, serving as the central medial landmark for trocar positioning (see Figure 4). The fasciae of the posterior abdominal wall are equally important. The thoracolumbar fascia, a thin fibrous layer, covers most of the aforementioned muscles (see Figure 4) and is divided into three sagittal layers: the anterior layer (marked *, in green, in Figure 3), which covers the quadratus lumborum, alongside the middle (marked **, in light blue, in Figure 3) and posterior (marked ***, in deep blue, in Figure 3) layers, which envelop the ES muscles and unite towards the lateral border of the ES group to form a strong raphe. Thereafter, the raphe is reached by the aforementioned anterior layer, forming the aponeurosis of the TA muscle at the lateral border of the quadratus lumborum [42] (see Figure 3). The transversalis fascia, which lines the deep surface of the TA muscle, and the psoas fascia, covering the psoas muscle, are also important anatomical features to be aware of (see Figure 3).

The Grynfeltt–Lesshaft triangle, or superior lumbar triangle (see Figure 4), is typically penetrated by the medial trocar during PRLA. This triangle is bounded laterally by the dorsal border of the IO muscle, medially by the lateral border of the ES muscles, and cranially by the 12th rib. Occasionally, the triangle assumes a square shape, when the angle between the 12th rib and the spinal muscles is covered by the serratus posterior inferior muscle. Within this triangle, the TA aponeurosis is directly covered by the latissimus dorsi muscle [32,43]. The Grynfeltt triangle is a weak point of the abdominal wall and is prone to herniation. The true weak point is the lateral half, where the TA aponeurosis is perforated by vessels and nerves, as the medial half is ventrally covered by the quadratus lumborum [44,45] (see Figure 3).

The Petit triangle, or inferior lumbar triangle (see Figure 4), is another area of weakness and potential herniation, observed when the latissimus dorsi muscle does not extend to the EO muscle. The boundaries of this triangle are the dorsal edge of the EO muscle laterally, the lateral free edge of the latissimus dorsi medially, and the posterior superior iliac crest bone inferiorly [43]. The Petit triangle, located inferior to the operative field, is generally not accessed during PRLA but may be inadvertently punctured when mounting an additional fourth trocar, if deemed necessary for retraction and achieving adequate exposure [31].

The blood supply to the posterior abdominal wall comes from the posterior ramifications of the intercostal arteries, lumbar arteries (arising from the posterior aspect of the abdominal aorta), and lateral sacral arteries (arising from the posterior divisions of the internal iliac arteries) [32]. Conversely, for the antero-lateral wall, perfusion is provided mainly by the superior and inferior epigastric and the deep circumflex iliac arteries, with some minor branches of the intercostal and lumbar vessels also present [42]. Venous drainage mirrors the arterial architecture. Generally, there is no risk of damaging these vascular structures during PRLA. However, awareness of their presence and course is important for honing one’s comprehensive anatomical understanding.

In contrast, the innervation of the posterior abdominal wall deserves special attention due to the risk of inadvertent injury during PRLA. The skin covering the back receives sensitive innervation from the spinal nerves, through their posterior rami. Thus, posterior cutaneous incisions, especially those more proximal to the midline, may be associated with temporary peri-incisional numbness, which is generally self-limiting and typically resolves on its own over time, as it would in any other area of the body [32].

Furthermore, the intercostal nerves, constituting the anterior rami of the spinal nerves, course along the caudal margins of the ribs, beneath the intercostal arteries. These mixed nerves provide somatic muscular branches to the intercostal muscles, as well as sensitive cutaneous branches (lateral and anterior) for the skin of the antero-lateral abdominal wall. Herein, ventral to the mid-axillary line, these lateral cutaneous branches of the intercostal nerves pierce the intercostal muscles and emerge through the parietal muscles to give sensitive cutaneous innervation [32].

Due to this anatomical conformation, the T12 intercostal nerve is actually subcostal, running along the caudal margin of the 12th rib and, thus, being particularly vulnerable during central and medial port placement. Initially, the subcostal T12 nerve emerges anterior to the quadratus lumborum and then travels between the TA and the IO muscles [42], only to subsequently follow the same distribution pattern as its counterparts, i.e., the other intercostal nerves. The lateral cutaneous branch of the T12 subcostal nerve emerges near the lateral edge of the quadratus lumborum, perforates the posterior parietal abdominal muscles, and becomes subcutaneous at the midpoint of the iliac crest, giving rise to several branches responsible for the cutaneous sensory innervation of the hip and gluteal regions [32]. Other relevant nerves include the iliohypogastric and ilioinguinal nerves (both L1), which run parallel and inferior to the subcostal nerve, and the lateral cutaneous nerve of the thigh (L2-3), which crosses the iliac crest. Caution is advised regarding horizontal incisions and large sutures, as these can entrap/transect nerves supplying motor innervation, increasing the risk of hernias/pseudo-hernias [32,42].

In summary, several surface anatomical landmarks are crucial for successful PRLA. The 12th rib serves as the superior limit of retroperitoneal access, while the posterior superior iliac crest defines the inferior limit. The ES muscles constitute the medial limit for port placement, and the posterior axillary line marks the lateral limit. It is worth noting that the adrenal glands themselves are encased by the 10th–12th ribs, providing some natural protection but also potentially limiting access. When accessing the retroperitoneum below the 12th rib, caution is advised in order to avoid injury to the T12 intercostal nerve, thus preventing abdominal wall relaxation or hypoesthesia. Ideally, during PRLA, the muscles of the abdominal wall that are traversed by the trocars should only include the latissimus dorsi, EO, IO, and TA. The paravertebral muscles, such as the ES and quadratus lumborum, should typically not be affected during the procedure, as they define the limit for medial trocar placement.

### 4.2. Retroperitoneal Topography

**Figure 5:**
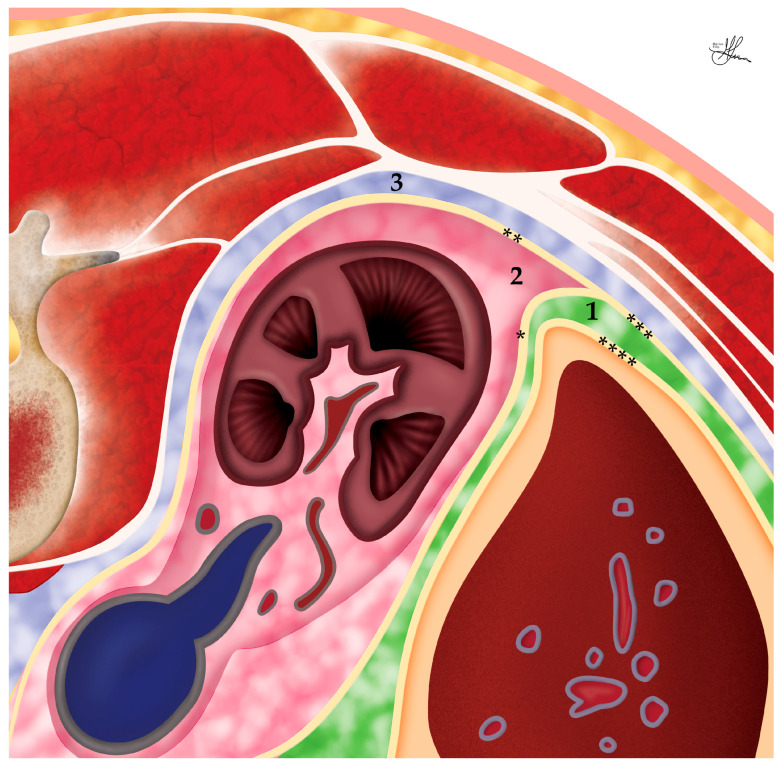
Illustration of the right retroperitoneal cavity, with highlighted fascial layers and compartmentation into retroperitoneal spaces: 1—anterior pararenal space (in green); 2—perirenal space (in pink); 3—posterior pararenal space (in blue); *—anterior perirenal fascia (Gerota’s fascia); **—posterior perirenal fascia (Zuckerkandl’s fascia); ***—lateroconal fascia; ****—posterior parietal peritoneum.

Understanding the anatomy of the retroperitoneal cavity is crucial for successful PRLA. For a proper management of the spatial relationships encountered during PRLA, this approach necessitates a thorough understanding of the retroperitoneum’s tripartite division into the following compartments:

1. The anterior pararenal space (marked 1, in green, in Figure 5), bordered anteriorly by the posterior parietal peritoneum (****) and posteriorly by the anterior perirenal fascia (Gerota’s fascia *). While not directly accessed during PRLA, it contains multiple vital structures: the ascending (right side) or descending (left side) colon laterally, and the duodenum, pancreas, and the mesenteric root of the small bowel centrally and medially. Although this posterior approach minimizes the risk of injury to these structures, their proximity underscores the importance of maintaining anatomical orientation throughout the procedure.
2. The perirenal space (marked 2, in pink, in Figure 5), bound within the perirenal fascia, contains the kidneys, adrenals, and proximal ureters, surrounded by perirenal fat. It is accessed by incising the perirenal fascia and represents the main workspace for PRLA.
3. The posterior pararenal space (marked 3, in blue, in Figure 5), delimitated between the posterior perirenal fascia (Zuckerkandl’s fascia **) anteriorly and the posterior abdominal wall musculature (quadratus lumborum, TA, and the thoracolumbar fascia) posteriorly. It is devoid of major organs and represents the first compartment accessed by the surgeon during PRLA, being then traversed sagittally to reach the more anterior perirenal workspace [32,46].

The retroperitoneal spaces are separated by avascular interfascial planes, a fact that surgeons can leverage to minimize bleeding during dissection. Moreover, these spaces extend through the posterior midline, adjoin the liver’s bare area and the hemidiaphragms bilaterally, and converge in the pelvis. This anatomical continuity creates potential routes for fluid or pathological dissemination between the thoracic and pelvic regions, a factor that must be considered in both surgical planning and postoperative care [47].

The fascial layers of the retroperitoneum play a crucial role in PRLA (see Figure 5). Gerota’s fascia (*), also known as the perirenal fascia, envelops both the kidney and the adrenal gland, forming the perirenal space. This fascia is divided into anterior (*) and posterior layers (**). The lateroconal fascia (***) extends from the transversalis fascia laterally [46]. These fascial planes are key to defining the surgical approach and guiding dissection during PRLA. The posterior approach used in PRLA enters the retroperitoneum behind Gerota’s fascia, providing a direct route to the adrenal gland.

Conversely, the surgical approach in PRLA leverages this anatomical arrangement to great effect. The procedure initiates with an incision through the abdominal wall, specifically lateral to the lumbo-iliac area. This entry point is strategically chosen to optimize access while minimizing the risk of iatrogenic injury. From this initial incision, the surgeon navigates through the posterior pararenal space before directly accessing the perirenal space through the perirenal fascia. A key advantage of this approach lies in the relative absence of vital structures within the posterior pararenal space. This anatomical “safe zone” significantly mitigates the risk of inadvertent visceral injury during the initial phases of the procedure. This feature is particularly advantageous when compared to traditional transperitoneal approaches, where the risk of injury to intra-abdominal organs is inherently higher [41].

The “backdoor” nature of PRLA thus represents not merely a change in surgical perspective but a fundamental shift in how surgeons interact with the retroperitoneal anatomy. By exploiting the natural avascular planes and relatively safe, yet confined, spaces of the retroperitoneum, PRLA offers a pathway to the adrenal glands that minimizes disruption to surrounding structures while maintaining a clear and direct route to the surgical target, constituting a promising technique for adrenal surgery, which balances direct access with minimized risk [1]. However, its successful implementation is contingent upon a comprehensive understanding of the retroperitoneal anatomy from a posterior perspective. This includes not only the spatial relationships of the adrenal glands and surrounding structures, but also the broader anatomical context of the retroperitoneal spaces.

The unique viewpoint required for PRLA challenges traditional surgical perspectives, necessitating a mental reorientation from anterior to posterior anatomical conceptualization. As surgical education and training evolve to incorporate this approach, PRLA may become increasingly prevalent in clinical practice. Further research into the long-term outcomes and potential applications of this technique will be crucial in defining its role in the surgical management of adrenal pathologies.

## 5. Achieving Adequate Surgical Exposure

To perform PRLA, standard laparoscopic equipment and instruments are required. This technique can be executed by any surgical department. Even so, standardizing the steps of the PRLA procedure is essential during the initial learning phase. This standardized approach is particularly useful at the beginning of the learning curve. However, dynamic procedural modifications may become necessary, depending on intraoperative findings. A skilled and experienced surgeon must be adaptable to varying situations. Experience gained from various minimally invasive procedures is invaluable, as techniques learned in one procedure can often be applied to another. Surgeons with prior laparoscopic experience can generally reduce the learning curve for PRLA significantly.

Achieving an adequate surgical exposure of the adrenals, within a well-defined, clean operative field, constitutes the main initial hurdle for clinicians beginning to implement this approach. In this section, we explore the factors involved in achieving this overarching operative goal, i.e., the positioning of the patient and surgical team, the technical aspects of trocar placement, and the principles involved in developing an adequate retroperitoneal workspace laparoscopically.

### 5.1. Surgical Team and Patient Positioning

The prone jackknife position, commonly used in PRLA, presents specific challenges. Although it generally does not pose significant issues for mechanical ventilation, it can increase the risk of complications such as accidental extubation, nerve injuries, and cardiovascular events (especially in the context of IVC manipulation). Anesthesia teams must be prepared for these complications and should be trained to manage emergencies in the prone position, including accidental extubation or cardiac arrest [48]. Positioning-related injuries, such as pressure-induced nerve damage or ophthalmic complications, must be meticulously prevented through careful positioning and regular monitoring. The risk of venous gas embolism, while low, should also be considered, particularly during CO_2_ insufflation [49].

**Figure 6:**
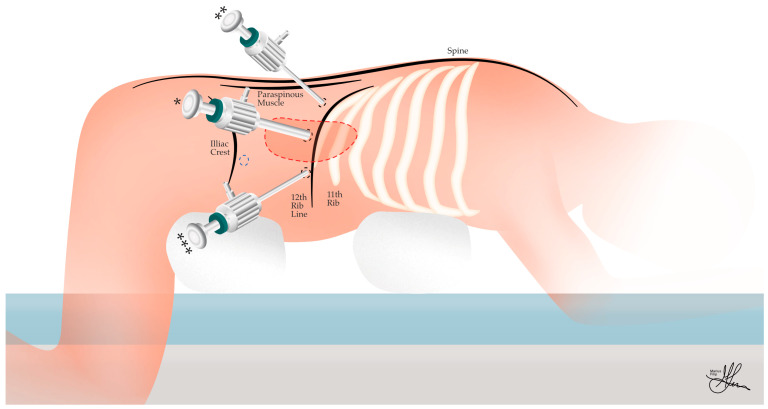
Prone jackknife positioning for posterior retroperitoneal laparoscopic adrenalectomy. *—Central 10 mm trocar; **—medial 5 mm trocar; ***—lateral 5 mm trocar; blue circle—optional additional 5 mm trocar, at times necessary for retraction.

Patients generally receive a prophylactic dose of preoperative antibiotics. A central venous catheter and an arterial line are usually required, especially for right-sided adrenalectomies, pheochromocytomas, or in the context of preexisting cardiovascular risks. In the supine position, sequential compression devices are applied for deep venous thrombosis prophylaxis, and general anesthesia induction and endotracheal intubation are achieved [50]. Thereafter, as seen in Figure 6, the patient is repositioned into a prone jackknife position. To achieve this, the surgical table must allow for adjustments to be made, to achieve an almost 90° hip flexion and to support bent knees, while ideally also allowing the abdomen to hang freely (Cloward Prone Positioner), reducing pressure on the retroperitoneal space [41]. Herein, it is important to position the superior anterior iliac crests correctly so that the abdominal wall is free and not restricted by hip supports, as the thighs are positioned with supported knees. Alternatively, a roll can be placed under the anterior iliac crests and another under the patient’s thorax, facilitating the movement of the abdominal viscera away from the retroperitoneum, which is vital for creating an adequate working space [15]. Adjustable supports attached to the operating table should be used to align with the patient’s body size.

The anesthesia team pads the patient’s face and uses a mirror on the table to monitor the endotracheal tube’s position. The table is set with little to no Trendelenburg tilt. The patient’s arms are flexed and pronated on arm boards, with elbows bent at 90°, and the hips and knees are also bent at 90°, with all pressure points adequately padded. The knee rest is lowered to reduce pressure on the knees and prevent posterior displacement of the hips, which could interfere with instrument manipulation. Attention to detail in this setup phase is crucial, as it facilitates the surgical procedure [15,41].

Overall, the jackknife position may prove beneficial for increasing the distance between trocars, facilitating the manipulation of instruments within the limited working space. Thus, with the hips flexed at a 90° angle to maximize the space between the 12th rib and the iliac crest, this position provides optimal exposure for port placement and surgical access in PRLA. Moreover, this position has several further favorable anatomical implications. The position causes the abdominal contents to fall anteriorly, creating more working space in the retroperitoneum. It also shifts the kidneys caudally, improving exposure of the adrenal glands, and moves the diaphragm cranially, potentially improving respiratory mechanics. Finally, the posterior abdominal wall is stretched in this position, facilitating port placement [41].

The surgical team consists of the surgeon, the scopist, and the scrub nurse. The surgeon and the scopist are positioned on the same side as the gland to be removed, while the scrub nurse is on the opposite side. The screen is positioned directly in front of the surgeon, at eye level. The surgeon utilizes both working ports, and the scopist is responsible for manipulating the camera throughout the procedure [41].

### 5.2. Trocar Placement Technique

Proper placement of trocars is essential in retroperitoneoscopic procedures. Incorrectly positioned trocars can complicate the surgery, cause discomfort and strain in the surgeon’s arms and back, increase anxiety in the surgical team as they struggle with instrument manipulation, and compromise patient safety. Marking anatomical landmarks prior to incision can be helpful for operative planning. As seen in Figure 6, we recommend identifying the lateral border of the paraspinous muscles, the inferior margin of the 12th rib, and the posterior superior iliac crest [24,51].

**Figure 7:**
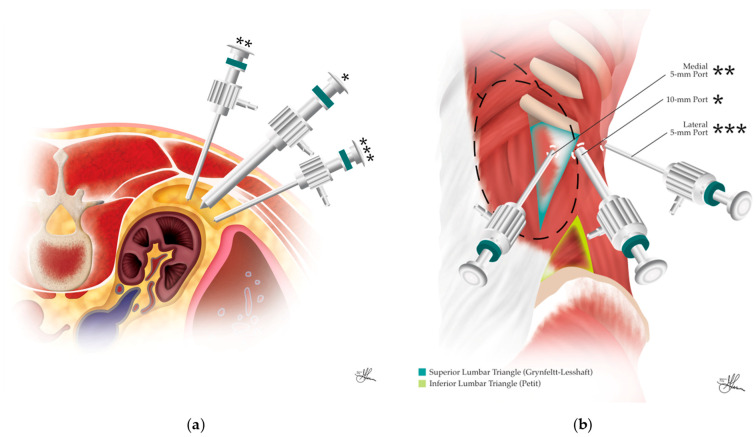
Comparative illustrations of trocar placement for posterior retroperitoneal laparoscopic adrenalectomy on the right side: (**a**) Axial section, prone position, superior view (from the head of the patient). (**b**) Coronal section, prone position, posterior perspective (from the surgeon’s perspective).

Access to the retroperitoneal space is typically gained just below the tip of the 12th rib. Key considerations during this step include avoiding injury to the pleura superiorly, identifying the correct fascial plane posterior to the kidney, and avoiding inadvertent entry into the peritoneum anteriorly. Subsequent ports are placed in a triangular configuration, with the central camera port (* in Figure 6 and Figure 7a,b) situated 1–2 cm below the 12th rib’s tip, and the two working ports placed medially (** in Figure 6 and Figure 7a,b), bordering the paraspinous muscles, and laterally (*** in Figure 6 and Figure 7a,b), near the posterior axillary line. An optional fourth port (blue circle in Figure 6) may be placed inferiorly for retraction if needed [24,41].

The initial 10 mm trocar insertion site is determined by palpating the 12th rib, identifying the tip of the rib, and creating a 1.5 cm transverse incision, one fingerbreadth caudally. This initial incision must admit one finger to help with blunt dissection and be able to fit a 10 mm trocar. The location of the first trocar is critical; the presence of thoracolumbar fascia rather than muscular fibers indicates an excessively medial approach (see Figure 7a). Even if the thoracolumbar fascia is encountered but the 10 mm trocar is passed just laterally to it, the superior lumbar triangle (of Grynfeltt–Lesshaft)—bound by the 12th rib superiorly, the IO muscle laterally, and the ES muscles medially (as seen in Figure 7b)—will still likely be traversed. In this thinnest area of the posterior abdominal wall, the musculo-fascial parietal anatomy, from superficial to deep, includes the latissimus dorsi muscle, EO muscle, and transversalis fascia. This region represents a potential site of reduced structural integrity in the posterior abdominal wall. While direct traversal of this triangle is not contraindicated, it necessitates meticulous closure to mitigate the risk of incisional hernia formation. Conversely, the optimal trajectory traverses the skin, subcutaneous adipose tissue, and EO, IO, and TA muscles, in that order, culminating in the penetration of the transversalis fascia to access the retroperitoneum, i.e., the posterior pararenal space (see Figure 5). Therefore, encountering the latissimus dorsi indicates that the incision is placed too supero-medial [24,32,41].

Access to the retroperitoneal space is accomplished by blunt dissection with a Kelly clamp or sharp dissection with scissors. Once retroperitoneal access is achieved, a finger sweep should be performed, with further blind blunt digital dissection extended medially and laterally, to allow for the creation of sufficient retroperitoneal space for the insertion of two more 5 mm work trocars. With an index finger inserted into the retroperitoneal space, guiding the insertion just lateral to the paraspinous muscles, approximately 3 cm below the 12th rib, the first 5 mm medial trocar is carefully positioned, angled cranially at 45 degrees to optimize access to the surgical field. Similarly, positioned 4–5 cm lateral to the initial incision, below the 11th rib, the second 5 mm lateral trocar is inserted. Finally, a larger 10–12 mm blunt trocar, equipped with an inflatable dissector balloon, is passed through the initial incision site and serves as the main working port for the procedure. Once all ports are successfully positioned, the surgeon establishes pneumoretroperitoneum by insufflating the retroperitoneal space with CO_2_ gas. The pressure is carefully maintained between 20 and 28 mmHg, creating and sustaining the necessary working space for the adrenalectomy in the relatively non-compliant retroperitoneal cavity [41,52].

Alternatively, following initial access, the creation of a sufficient working space within the retroperitoneum is achieved through either digital dissection or the direct deployment of a balloon dissector. After the first 10 mm trocar is inserted and secured, pneumoretroperitoneum is then established from the beginning, and a 30° laparoscope is passed, aiding with further blunt dissection. Finally, in the same positions as described above, but under direct laparoscopic visualization, the two additional 5 mm trocars are inserted. The lateral port is placed a minimum of 3 cm from the central port to minimize instrument interference while maintaining triangulation [25]. The resulting trocar configuration forms a horizontal alignment, which allows for dynamic adaptation of the port function intraoperatively, facilitating optimal visualization and instrument maneuverability in response to individual anatomical variations and specific surgical challenges.

### 5.3. Workspace Development

Creating an adequate retroperitoneal workspace can be the most challenging task in PRLA. As stated above, patient positioning is essential for allowing this to occur. With the abdominal contents hanging freely, the retroperitoneal cavity is expanded, in the prone jackknife position. However, the working space can differ between sides. The right side often provides a slightly more confined working area due to the presence of the liver, while the left side may offer more room for manipulation, particularly in the upper pole of the gland. Another key difference lies in the surrounding structures that must be navigated. On the right, the liver serves as an important landmark and potential obstacle, while on the left, the spleen and pancreatic tail are the primary structures of concern. The right-sided procedure often requires more careful retraction of the liver to obtain adequate exposure [53].

Regardless of the trocar placement technique, initially, a 10 mm 30° endoscope is introduced through the middle trocar. This larger scope is used to begin the creation of the retroperitoneal space. The surgeon employs a combination of blunt and sharp dissection techniques to carefully expand this area, providing the necessary room for the procedure. Once this initial space is established, the surgeon transitions to a smaller, more maneuverable instrument. The 10 mm scope is replaced with a 5 mm 30-degree scope, which is then inserted through the medial trocar. This change allows for greater flexibility and improved visualization in the confined retroperitoneal space [52].

With the 5 mm scope in place, the surgeon focuses on expanding the retroperitoneal space beneath the diaphragm. This is achieved by applying downward pressure on the fatty tissue in this area. The goal is to create sufficient working space while maintaining careful control to avoid injury to surrounding structures.

A key step in this process is the identification of and entry into Gerota’s fascia. This fascial layer encapsulates the kidney and the adrenal gland, and its careful dissection is crucial for accessing the adrenal gland. As the dissection progresses, the surgeon works to identify the superior pole of the kidney. This anatomical landmark serves as a critical reference point during the procedure. Locating the superior pole of the kidney not only helps orient the surgeon within the retroperitoneal space but also guides the subsequent dissection towards the adrenal gland, which is typically located just above and medial to this point. This meticulous approach to creating and navigating the retroperitoneal space sets the stage for the subsequent steps of the adrenalectomy, ensuring optimal visibility and access to the adrenal gland while minimizing the risk of injury to surrounding structures [41,52].

As the dissection progresses deeper, the surgeon encounters the paraspinous muscles and, ultimately, visualizes the adrenal gland. The anatomical landscape differs slightly between the right and left sides. On the right, the IVC serves as a crucial anatomical reference point, and it is not uncommon to glimpse the liver through the translucent posterior peritoneum. In contrast, when operating on the left side, the spleen or the posterior gastric wall may be visible through the thin peritoneal lining [41]. These surrounding structures provide important spatial orientation during the procedure, guiding the surgeon’s approach to the adrenal gland.

## 6. Optimal Approach to Adrenal Dissection

The PRLA technique, while similar in overall approach, presents distinct challenges and considerations depending on whether the left or right adrenal gland is being addressed. Understanding these differences is crucial for optimal surgical outcomes.

For both sides, the initial steps are similar: patient positioning in a prone jackknife position, trocar placement, and creation of the retroperitoneal space. Dissection commences at the inferior pole of the adrenal gland, progressing medially. Small arterial vessels feeding the gland are divided using electrocautery, i.e., vascular sealing devices. It is crucial to maintain the superior attachments of the adrenal gland intact at this stage, allowing it to “hang” and facilitating further dissection [52]. This strategy minimizes manipulation of the relatively fragile adrenal tissue. However, as the dissection progresses, key anatomical differences become apparent.

**Figure 8:**
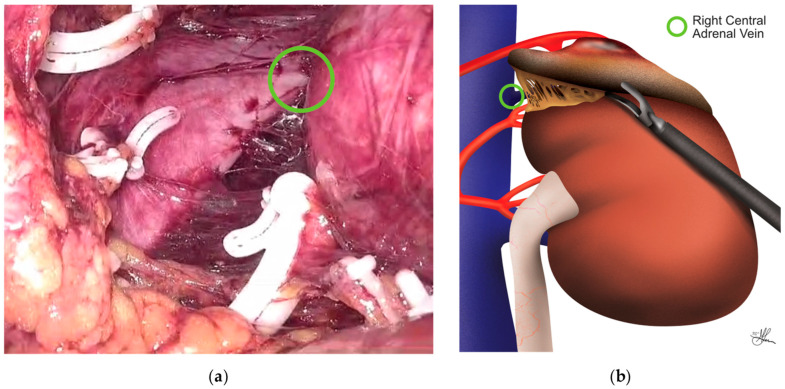
Right posterior retroperitoneal laparoscopic adrenalectomy, with the characteristic junction between the central right adrenal vein and the inferior vena cava highlighted by the green circle: (**a**) live surgery image; (**b**) illustration.

On the right side, dissection begins lateral and posterior to the right lobe of the liver, progressing medially until the lateral aspect of the IVC is visualized. The right adrenal vein, a critical structure, is typically found posterolateral to the IVC, often close to the diaphragm. It courses medially to drain directly into the IVC (see Figure 8a,b). The surgeon must exercise extreme caution when dissecting in this area, as inadvertent injury to the IVC can lead to significant bleeding. The high insufflation pressure used in PRLA often flattens the IVC, causing it to lose its tubular appearance and appear as a white line along its lateral border [24,25,41,54].

**Figure 9:**
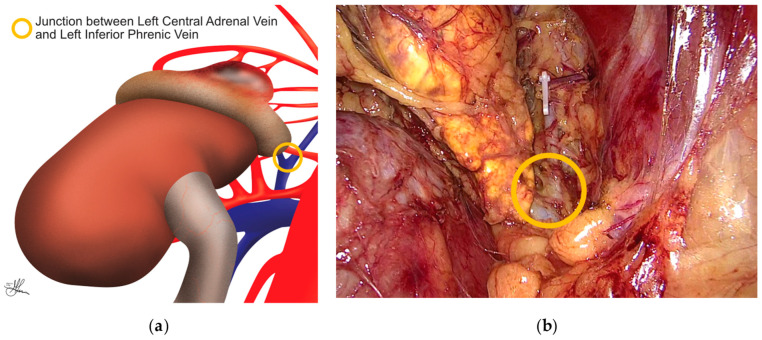
Left posterior retroperitoneal laparoscopic adrenalectomy, with the characteristic junction between the central left adrenal vein and the left inferior phrenic vein highlighted by the yellow circle: (**a**) illustration; (**b**) live surgery image.

In contrast, left-sided adrenalectomy presents a different set of anatomical relationships. Dissection on the left begins laterally, in the plane between the kidney and the adrenal gland, behind the spleen. As dissection continues medially, the surgeon must be aware of the proximity of the pancreatic tail and take care to avoid injury. The left adrenal vein is typically found medial to the upper pole of the kidney, draining inferiorly into the left renal vein, usually within a common venous trunk formed by its junction with the left inferior phrenic vein superiorly (see Figure 9a,b), rather than directly into the IVC as on the right side. This anatomical arrangement often makes the left adrenal vein easier to identify and control [24,25,41,54].

Overall, in terms of the adrenal vein management, while both sides require careful isolation and ligation, the right adrenal vein is often shorter and more challenging to dissect due to its proximity to the IVC. On the left, the adrenal vein is typically longer and more easily isolated, but care must be taken not to injure the left renal vein during dissection. Thus, on both sides, once identified, the adrenal vein should be circumferentially dissected for about 1 cm and ligated using an advanced sealing device or clips. This step is crucial for maintaining hemostasis and preventing inadvertent bleeding. After securing the adrenal vein, dissection continues laterally and superiorly. The adrenal vein’s stump can be used as a handle for manipulation, minimizing direct grasping of the friable adrenal tissue. The phrenic vein may be divided if needed but can often be preserved. For both sides, small arterial branches are encountered and coagulated throughout the dissection. However, the right side may require more meticulous dissection due to the presence of small arteries crossing the IVC posteriorly [24,25,41,54].

Adrenal gland dissection is completed by freeing the gland from its remaining attachments to the paraspinous muscles and peritoneum. Throughout this process, it is important to avoid grasping the actual adrenal parenchyma, which is easily fractured. Once fully mobilized, the specimen is placed in a retrieval bag and extracted through the middle trocar site, which may occasionally need minimal enlargement. Before closure, the insufflation pressure is reduced to 8–12 mmHg to check for any venous bleeding that might have been tamponaded by the high CO_2_ pressure. The adrenal bed is irrigated, and meticulous hemostasis is achieved. Lastly, the trocar insertion sites must be checked for bleeding after trocar removal, and the incisions must be closed appropriately. The fascia of the 10 mm trocar site should ideally be closed with a figure-of-eight suture, and the skin of all port sites with a subcuticular stitch [41,54].

Importantly, throughout the procedure, vigilance must be maintained against potential complications such as pneumothorax or peritoneal breach, which could lead to inadvertent injury of adjacent organs, i.e., the spleen, pancreas, or liver. A chest X-ray in the recovery area can ensure that no pneumothorax has occurred.

This systematic approach to PRLA, coupled with the surgeon’s growing experience in identifying the bright yellow appearance of the adrenal cortex amid retroperitoneal fat, ensures safe and effective adrenal gland removal while minimizing the risk of complications. The high-pressure pneumoretroperitoneum not only creates additional working space in this confined area but also helps to tamponade minor bleeding from small arterioles, contributing to the procedure’s efficiency and safety.

## 7. Surgical Tips and Tricks

PRLA has emerged as a safe, fast, and effective procedure with a low complication rate. However, mastering this technique requires attention to several key aspects and potential challenges. This section provides a comprehensive overview of essential tips and tricks to optimize the surgical approach and outcomes.

One of the initial concerns with PRLA was the ability to create adequate working space in the retroperitoneum using traditional insufflation pressures. Contrary to early apprehensions about higher insufflation pressures decreasing venous return and causing hypotension due to IVC compression, research has demonstrated that patients maintain normal cardiac output and filling pressures with elevated retroperitoneal insufflation levels. Studies comparing intra- and extra-thoracic IVC pressures during laparoscopic cholecystectomy and PRLA revealed that while cardiac filling decreased with intraperitoneal insufflation pressures above 15 mmHg, it remained stable with retroperitoneal insufflation pressures exceeding this threshold. Consequently, surgeons can safely use higher insufflation pressures (up to 28–30 mmHg) to create sufficient working space and achieve a relatively bloodless operative field [54].

In obese patients (BMI > 35), increasing retroperitoneal pneumo-insufflation up to 30 mmHg can help establish an adequate working space. However, patients with a BMI > 45 may not be ideal candidates for PRLA. In these cases, the compression of the retroperitoneum by abdominal organs in the prone position can make it extremely challenging to create sufficient space, even with high insufflation pressures. Some studies have reported the need to convert to a transabdominal approach in patients with BMI > 45 due to this issue. While obesity does not preclude PRLA, it does make the procedure more technically challenging and time-consuming, underscoring the importance of careful patient selection and surgeon experience [41].

Correct trocar placement is crucial for safe and comfortable gland dissection. The trocars should be placed at an acute angle towards the adrenal. This becomes even more critical in obese patients or in males with thick muscle walls. In rare instances of trocar misplacement causing pleural tears, the procedure can often be continued by sealing the leakage with a blunt-tipped trocar with a balloon and inserting a pleural drain until the end of the operation. If the peritoneal cavity is inadvertently entered during the procedure, surgery can typically still be completed using the posterior retroperitoneoscopic approach without additional intervention, as there is no compression of the retroperitoneum by the abdominal viscera [55].

Identifying the upper pole of the kidney is paramount, as it provides the critical anatomical landmark for safe progression of the procedure [56,57]. Dissection should always proceed from “lateral to medial” and from “bottom to top”, commencing at the lower pole of the suprarenal gland. Right-sided dissection should follow a clockwise movement from 3 to 9 o’clock, while on the left side it should be counterclockwise from 9 to 3 o’clock. This approach helps avoid injury to the renal vessels [17]. The left adrenal gland’s position, falling in front of the superior part of the left kidney’s anterior surface, necessitates extended kidney mobilization for complete gland resection. This can be achieved by applying pressure on the kidney’s upper pole with a non-traumatic grasper.

Retroperitoneal fatty tissue can significantly obscure the surgical field, especially in obese patients or those with Cushing’s syndrome. Removing this tissue, often possible through suctioning, especially around the superior renal pole and its ipsilateral adrenal, can help expose crucial anatomical landmarks [58]. This step is essential for identifying key structures and facilitating safe dissection [54].

Vascular considerations are critical in PRLA. The right adrenal vein, typically short and positioned behind the gland and in front of the IVC, can be challenging to locate and dissect. Gently lifting the right adrenal from the IVC can create a small fold on the IVC’s dorsal surface, helping to locate the vein. When dissecting near the IVC, using a “pinch and pull” technique on fatty tissue can help ensure that the vein is not inadvertently grasped. Ligating the medial adrenal arteries crossing the vena cava in a retro-caval position may aid in exposing the IVC [17].

While significant bleeding is rare in PRLA, it can usually be controlled by the 20–25 mmHg pressure and energy devices. Applying compression with gauze is often the fastest and simplest way to control bleeding if it occurs. Clips can be used cautiously if needed, but they are rarely necessary. The high CO_2_ pressure in the retroperitoneal space helps to tamponade small-vessel bleeding, contributing to the procedure’s safety.

Managing potential complications is an essential aspect of PRLA. Pneumothorax can occur if the pleural space is violated during trocar placement or dissection, with reported rates between <1 and 3.4%. Palpable subcutaneous emphysema may also occur but is typically asymptomatic and does not alter the hospital course. Rarely, a fourth trocar may be placed below the line of the first trocars (see Figure 6). This additional port may be useful in placing a vascular clamp to control bleeding in the event of a major vascular injury, or for kidney retraction in obese patients [54].

During the procedure, keeping the left adrenal vein grasped with a non-traumatic grasper after ligation can help mobilize the gland throughout the remaining procedure, without capsule rupture [59]. This technique is not applicable to the short right adrenal vein. When dissecting the entire gland, dissecting from “top to bottom” may occasionally help to mobilize it completely [54,55].

PRLA is a technically demanding procedure that requires a thorough understanding of the retroperitoneal anatomy. With experience and proper technique, it offers a safe and effective approach for adrenal surgery, even in challenging cases. However, careful patient selection, especially regarding BMI, remains crucial for optimal outcomes. As with any advanced laparoscopic procedure, the learning curve can be significant, and surgeons should ideally gain experience under the guidance of an experienced mentor before attempting complex cases independently.

## 8. Conclusions

In conclusion, PRLA represents a significant advancement in the field of adrenal surgery, offering numerous benefits over traditional open approaches. Its adoption has been driven by the clear advantages of reduced postoperative pain, shorter hospital stays, faster recovery times, and comparable long-term outcomes. However, the expertise of the surgical team and the specific characteristics of each case must guide the choice of surgical approach, as PRLA is not without limitations. Obesity, large tumors, and suspected malignancies may pose significant challenges or contraindications. With careful patient selection, meticulous perioperative management, and a multidisciplinary approach, PRLA provides an effective and minimally invasive option for treating adrenal tumors, while open surgery continues to play a crucial role in managing more complex cases. As the field of laparoscopic adrenal surgery continues to evolve, further research and advancements in techniques and technology will likely expand the indications and improve outcomes for patients undergoing this innovative procedure.

## Data Availability

Data are available upon request.

## References

[1] Uludağ M., Aygün N., İşgör A. (2020). Surgical Indications and Techniques for Adrenalectomy. Sisli Etfal Hastan. Tip. Bul..

[2] Prager G., Heinz-Peer G., Passler C., Kaczirek K., Schindl M., Scheuba C., Niederle B. (2002). Surgical Strategy in Adrenal Masses. Eur. J. Radiol..

[3] Matsuda T. (2017). Laparoscopic Adrenalectomy: The “gold Standard” When Performed Appropriately. BJU Int..

[4] Go H., Takeda M., Takahashi H., Imai T., Tsutsui T., Mizusawa T., Nishiyama T., Morishita H., Nakajima Y., Sato S. (1993). Laparoscopic Adrenalectomy for Primary Aldosteronism: A New Operative Method. J. Laparoendosc. Surg..

[5] Higashihara E., Tanaka Y., Horie S., Aruga S., Nutahara K., Homma Y., Minowada S., Aso Y. (1992). A case report of laparoscopic adrenalectomy. Nihon Hinyokika Gakkai Zasshi.

[6] Gagner M., Lacroix A., Bolté E. (1992). Laparoscopic Adrenalectomy in Cushing’s Syndrome and Pheochromocytoma. N. Engl. J. Med..

[7] Wu C.-T., Chiang Y.-J., Chou C.-C., Liu K.-L., Lee S.-H., Chang Y.-H., Chuang C.-K. (2005). Comparative Study of Laparoscopic and Open Adrenalectomy. Chang. Gung Med. J..

[8] Al-Jalabneh T., Al-Shawabkeh O., Al-Gwairy I., Abu-Zeitoun O., Al-Njadat I., Al-Soudi M., Zarour A. (2021). Laparoscopic Versus Open Adrenalectomy: A Retrospective Comparative Study. Med. Arch..

[9] Guerrieri M., Campagnacci R., De Sanctis A., Baldarelli M., Coletta M., Perretta S. (2008). The Learning Curve in Laparoscopic Adrenalectomy. J. Endocrinol. Investig..

[10] Guidelines for the Minimally Invasive Treatment of Adrenal Pathology—A SAGES Publication. SAGES. https://www.sages.org/publications/guidelines/guidelines-for-the-minimally-invasive-treatment-of-adrenal-pathology/.

[11] Kapoor A., Morris T., Rebello R. (2011). Guidelines for the Management of the Incidentally Discovered Adrenal Mass. Can. Urol. Assoc. J..

[12] Tanaka M., Ono Y., Matsuda T., Terachi T., Suzuki K., Baba S., Hara I., Hirao Y., Urological Laparoscopic Surgery Guideline Committee, Japanese Society of Endourology and ESWL (2009). Guidelines for Urological Laparoscopic Surgery. Int. J. Urol..

[13] Maccora D., Walls G., Sadler G., Mihai R. (2017). Bilateral Adrenalectomy: A Review of 10 Years’ Experience. Ann. R. Coll. Surg. Engl..

[14] Mercan S., Seven R., Ozarmagan S., Tezelman S. (1995). Endoscopic Retroperitoneal Adrenalectomy. Surgery.

[15] Walz M.K., Peitgen K., Hoermann R., Giebler R.M., Mann K., Eigler F.W. (1996). Posterior Retroperitoneoscopy as a New Minimally Invasive Approach for Adrenalectomy: Results of 30 Adrenalectomies in 27 Patients. World J. Surg..

[16] Mihai R. (2019). Bilateral Adrenalectomy—Simultaneous or Delayed?. Laparosc. Surg..

[17] Alesina P.F. (2019). Retroperitoneal Adrenalectomy—Learning Curve, Practical Tips and Tricks, What Limits Its Wider Uptake. Gland. Surg..

[18] Walz M.K., Alesina P.F., Wenger F.A., Deligiannis A., Szuczik E., Petersenn S., Ommer A., Groeben H., Peitgen K., Janssen O.E. (2006). Posterior Retroperitoneoscopic Adrenalectomy—Results of 560 Procedures in 520 Patients. Surgery.

[19] Chai Y.J., Yu H.W., Song R.-Y., Kim S., Choi J.Y., Lee K.E. (2019). Lateral Transperitoneal Adrenalectomy Versus Posterior Retroperitoneoscopic Adrenalectomy for Benign Adrenal Gland Disease: Randomized Controlled Trial at a Single Tertiary Medical Center. Ann. Surg..

[20] Jiang Y.-L., Qian L.-J., Li Z., Wang K.-E., Zhou X.-L., Zhou J., Ye C.-H. (2020). Comparison of the Retroperitoneal versus Transperitoneal Laparoscopic Adrenalectomy Perioperative Outcomes and Safety for Pheochromocytoma: A Meta-Analysis. BMC Surg..

[21] Alemanno G., Bergamini C., Prosperi P., Valeri A. (2017). Adrenalectomy: Indications and Options for Treatment. Updates Surg..

[22] Lal G., Clark O.H., Brunicardi F.C., Andersen D.K., Billiar T.R., Dunn D.L., Kao L.S., Hunter J.G., Matthews J.B., Pollock R.E. (2019). Thyroid, Parathyroid, and Adrenal. Schwartz’s Principles of Surgery.

[23] Mihai I., Boicean A., Teodoru C.A., Grigore N., Iancu G.M., Dura H., Bratu D.G., Roman M.D., Mohor C.I., Todor S.B. (2023). Laparoscopic Adrenalectomy: Tailoring Approaches for the Optimal Resection of Adrenal Tumors. Diagnostics.

[24] Fuentes M.B., Keat C.W., Lomanto D., Chen W.T.-L., Fuentes M.B. (2023). Laparoscopic Adrenalectomy: Retroperitoneal Approach. Mastering Endo-Laparoscopic and Thoracoscopic Surgery: ELSA Manual.

[25] McCoy K., Valdez C., Gibson C.E. (2022). Retroperitoneoscopic Adrenalectomy: Indications and Technical Considerations. Laparosc. Surg..

[26] Lenders J.W.M., Duh Q.-Y., Eisenhofer G., Gimenez-Roqueplo A.-P., Grebe S.K.G., Murad M.H., Naruse M., Pacak K., Young W.F., Endocrine Society (2014). Pheochromocytoma and Paraganglioma: An Endocrine Society Clinical Practice Guideline. J. Clin. Endocrinol. Metab..

[27] Arezzo A., Bullano A., Cochetti G., Cirocchi R., Randolph J., Mearini E., Evangelista A., Ciccone G., Bonjer H.J., Morino M. (2018). Transperitoneal versus Retroperitoneal Laparoscopic Adrenalectomy for Adrenal Tumours in Adults. Cochrane Database Syst. Rev..

[28] Berruti A., Baudin E., Gelderblom H., Haak H.R., Porpiglia F., Fassnacht M., Pentheroudakis G., ESMO Guidelines Working Group (2012). Adrenal Cancer: ESMO Clinical Practice Guidelines for Diagnosis, Treatment and Follow-Up. Ann. Oncol..

[29] Fassnacht M., Dekkers O., Else T., Baudin E., Berruti A., de Krijger R., Haak H., Mihai R., Assie G., Terzolo M. (2018). European Society of Endocrinology Clinical Practice Guidelines on the Management of Adrenocortical Carcinoma in Adults, in Collaboration with the European Network for the Study of Adrenal Tumors. Eur. J. Endocrinol..

[30] Gaujoux S., Mihai R., joint working group of ESES and ENSAT (2017). European Society of Endocrine Surgeons (ESES) and European Network for the Study of Adrenal Tumours (ENSAT) Recommendations for the Surgical Management of Adrenocortical Carcinoma. Br. J. Surg..

[31] Callender G.G., Kennamer D.L., Grubbs E.G., Lee J.E., Evans D.B., Perrier N.D. (2009). Posterior Retroperitoneoscopic Adrenalectomy. Adv. Surg..

[32] Caroço T.V., Costa Almeida C.E., Eduardo Costa Almeida C. (2023). Anatomy of the Adrenal Gland. Posterior Retroperitoneoscopic Adrenalectomy: Indications, Technical Steps and Outcomes.

[33] Ceccato F., Scaroni C., Boscaro M., Belfiore A., LeRoith D. (2016). The Adrenal Glands. Principles of Endocrinology and Hormone Action.

[34] Van Slycke S., Van Den Heede K., Vandenwyngaerden E.-A., Shifrin A.L., Raffaelli M., Randolph G.W., Gimm O. (2021). Adrenal Glands: Anatomy, Physiology, and Pathophysiology. Endocrine Surgery Comprehensive Board Exam Guide.

[35] Clark O.H., Duh Q.-Y., Kebebew E., Gosnell J.E., Shen W.T. (2016). Textbook of Endocrine Surgery.

[36] Avisse C., Marcus C., Patey M., Ladam-Marcus V., Delattre J.F., Flament J.B. (2000). Surgical Anatomy and Embryology of the Adrenal Glands. Surg. Clin. North. Am..

[37] Feigelson B.J., Skandalakis L.J. (2021). Adrenal Glands. Surgical Anatomy and Technique: A Pocket Manual.

[38] Themes U.F.O. The Adrenal Glands. Thoracic Key. https://thoracickey.com/the-adrenal-glands/.

[39] Cesmebasi A., Du Plessis M., Iannatuono M., Shah S., Tubbs R.S., Loukas M. (2014). A Review of the Anatomy and Clinical Significance of Adrenal Veins. Clin. Anat..

[40] Megha R., Wehrle C.J., Kashyap S., Leslie S.W. (2024). Anatomy, Abdomen and Pelvis: Adrenal Glands (Suprarenal Glands). StatPearls.

[41] Costa Almeida C.E., Eduardo Costa Almeida C. (2023). Technical Steps of Posterior Retroperitoneoscopic Adrenalectomy. Posterior Retroperitoneoscopic Adrenalectomy: Indications, Technical Steps and Outcomes.

[42] Flynn W., Vickerton P. (2024). Anatomy, Abdomen and Pelvis: Abdominal Wall. StatPearls.

[43] Loukas M., El-Zammar D., Shoja M., Tubbs R.S., Zhan L., Protyniak B., Krutoshinskaya Y. (2008). The Clinical Anatomy of the Triangle of Grynfeltt. Hernia J. Hernias Abdom. Wall Surg..

[44] Armstrong O., Hamel A., Grignon B., NDoye J.M., Hamel O., Robert R., Rogez J.M. (2008). Lumbar Hernia: Anatomical Basis and Clinical Aspects. Surg. Radiol. Anat..

[45] Orcutt T.W. (1971). Hernia of the Superior Lumbar Triangle. Ann. Surg..

[46] Standring S. (2020). Gray’s Anatomy: The Anatomical Basis of Clinical Practice.

[47] Tirkes T., Sandrasegaran K., Patel A.A., Hollar M.A., Tejada J.G., Tann M., Akisik F.M., Lappas J.C. (2012). Peritoneal and Retroperitoneal Anatomy and Its Relevance for Cross-Sectional Imaging. Radiographics.

[48] Raslau D., Bierle D.M., Stephenson C.R., Mikhail M.A., Kebede E.B., Mauck K.F. (2020). Preoperative Cardiac Risk Assessment. Mayo Clin. Proc..

[49] Edgcombe H., Carter K., Yarrow S. (2008). Anaesthesia in the Prone Position. Br. J. Anaesth..

[50] Feltracco P., Barbieri S., Carron M., Eduardo Costa Almeida C. (2023). Anesthesia in Posterior Retroperitoneoscopic Approach. Posterior Retroperitoneoscopic Adrenalectomy: Indications, Technical Steps and Outcomes.

[51] Themes U.F.O. Minimally Invasive Retroperitoneal Adrenalectomy. Abdominal Key. https://abdominalkey.com/minimally-invasive-retroperitoneal-adrenalectomy/.

[52] Cayo A.K., Wang T.S. (2013). Laparoscopic Adrenalectomy: Retroperitoneal Approach. Curr. Surg. Rep..

[53] Themes U.F.O. Posterior Retroperitoneoscopic Adrenalectomy. Oncohema Key. https://oncohemakey.com/posterior-retroperitoneoscopic-adrenalectomy/.

[54] Walz M.K., Linos D., Van Heerden J.A. (2005). Posterior Retroperitoneoscopic Adrenalectomy. Adrenal Glands.

[55] Perrier N.D., Kennamer D.L., Bao R., Jimenez C., Grubbs E.G., Lee J.E., Evans D.B. (2008). Posterior Retroperitoneoscopic Adrenalectomy: Preferred Technique for Removal of Benign Tumors and Isolated Metastases. Ann. Surg..

[56] Grosso A.A., Di Maida F., Tellini R., Mari A., Sforza S., Masieri L., Carini M., Minervini A. (2021). Robot-Assisted Partial Nephrectomy with 3D Preoperative Surgical Planning: Video Presentation of the Florentine Experience. Int. Braz. J. Urol..

[57] Grosso A.A., Di Maida F., Lambertini L., Cadenar A., Coco S., Ciaralli E., Salamone V., Vittori G., Tuccio A., Mari A. (2024). Three-Dimensional Virtual Model for Robot-Assisted Partial Nephrectomy: A Propensity-Score Matching Analysis with a Contemporary Control Group. World J. Urol..

[58] Simone G., Anceschi U., Tuderti G., Misuraca L., Celia A., De Concilio B., Costantini M., Stigliano A., Minisola F., Ferriero M. (2019). Robot-Assisted Partial Adrenalectomy for the Treatment of Conn’s Syndrome: Surgical Technique, and Perioperative and Functional Outcomes. Eur. Urol..

[59] Ferriero M., Iannuzzi A., Bove A.M., Tuderti G., Anceschi U., Misuraca L., Brassetti A., Mastroianni R., Guaglianone S., Leonardo C. (2024). Adrenalectomy for Metastasis: The Impact of Primary Histology on Survival Outcome. Cancers.

